# Effective prevention of early resignation of newly graduated nurses: a transactional analysis

**DOI:** 10.1186/s12912-024-02385-y

**Published:** 2024-10-08

**Authors:** Masanori Ogawa, Ryusuke Ae, Teppei Sasahara, Keiko Omi

**Affiliations:** 1 Health Service Center, Jichi Medical University, 3311-1, Yakushiji, Shimostuke, Tochigi, 329-0498 Japan; 2 Division of Public Health, Center for Community Medicine, Jichi Medical University, 3311-1, Yakushiji, Shimostuke, Tochigi, 329-0498 Japan; 3 Division of Infectious Disease, Department of Infection and Immunity, Jichi Medical University, 3311-1, Yakushiji, Shimostuke, Tochigi, 329-0498 Japan; 4 Nursing Department, Jichi Medical University Hospital, 3311-1, Yakushiji, Shimostuke, Tochigi, 329-0498 Japan

**Keywords:** Newly graduated nurses, Transactional analysis, Interpersonal relationship, Trait-anxiety, Personality

## Abstract

**Background:**

New employees often exhibit stress reactions to changes in their environment and some of these may result in resignations. Employees in medical institutions are no exceptions. Considering the shortage of nurses in Japan, countermeasures are needed against the resignation of newly graduated nurses. Many studies have indicated that multifaced factors affect the resignation of newly graduated nurses. Even though individual characteristics are important factors in this regard, training and support for newly graduated nurses do not focus on the nurses’ characteristics. The purpose of this study is to examine the characteristics influencing the early resignation of newly graduated nurses and suggest ways to support them and prevent their early resignation based on their characteristics.

**Methods:**

With the approval of the Ethics Committee, various characteristics of 353 newly graduated nurses (personality, interpersonal relationships, trait anxiety, nurse orientation, desire to be a nurse, and self-proclaimed academic ability) were assessed using a transactional analysis, the State-Trait Anxiety Inventory, and self-reporting numerical rating scales. The characteristics of those who resigned within 1 year (case group) and those who did not (control group) were compared using chi-square test and logistic regression analysis.

**Results:**

Our sample of 353 participants included 32 nurses from the case group and 304 nurses from the control group. Most participants showed similar personality traits. However, the control group had a significantly lower percentage with negative self, strong trait anxiety, negative self and strong trait anxiety, and low orientation compared to the case group. Our logistic regression analysis showed that strong trait anxiety and low nurse orientation are significantly related with the early resignation of nurses.

**Conclusions:**

The early resignation of newly graduated nurses may be prevented by understanding their characteristics at the time of gaining employment and implementing early intervention programs, such as education programs to reduce anxiety, and helping those with strong trait anxiety and low nurse orientation to find meaning in work.

## Background

Newly graduated employees often exhibit stress reactions to significant changes in their environment [1] and are disproportionately affected by mental health challenges [2]. Employees in medical institutions are no exceptions. Some newly graduated nurses feel highly stressed after gaining employment and seek resignation [3, 4].

Considering the shortage of healthcare workers, particularly nurses and midwives, both globally [5–7] and in Japan [8, 9], countermeasures are needed to prevent the resignation of newly graduated nurses.

Many studies have examined the newly graduated nurses’ early resignation in Japan and other countries. Kashiwada [10] reviewed some reports published from 2001 to 2017 in Japan on why newly graduated nurses resign soon after gaining employment, to find the following reasons: nurses find the working environment different from what they had expected; their skills acquired at nursing schools/colleges prove insufficient for their expected jobs; they find it difficult to get al.ong well with seniors; they do not fit in well both mentally and physically; and they lose interest in the work of nursing itself. In studies covering five hospitals in Japan, Imai [11] found that colleagues and seniors are the key issues to be considered in preventing the early resignation of newly graduated nurses. Tominaga [9] reported that effort, subjective health status, and having role models are important factors influencing the newly graduated nurses’ resignation. According to a report in the US, the causes of newly graduated nurses’ resignation are stressful working conditions, lack of leadership and supervision, and understaffed facilities [12]. In a review of 21 studies published from 2011 to 2022, Lyu [13] found that the main factors affecting newly graduated nurses’ resignation were demographic (age, educational level, year of experience, professional title, employment status, health status, shift, and hospital location and size), supervisor and peer support, challenges in workplace, cognitive and affective response to work, work environment (collegial nurse-physician relations, insufficient staffing level, person-work environment fit), gender stereotype, autonomous motivation, having role models, and resilience. Thus, multifaceted factors affect the resignation of newly graduated nurses.

According to the National Institute of Safety and Health (NIOSH) [14], job stress arises when job requirements do not match workers’ capabilities, resources, or needs. However, individual (age, sex, marital status, character, self-estimation, etc.), non-work-related (family demands), and buffering (social support from supervisors, coworkers, and family) factors also engender stress reactions [15]. Therefore, individual characteristics are also important factors affecting the resignation of newly graduated nurses.

Various hospitals conduct training and support programs for newly graduated nurses to prevent them from quitting soon after gaining employment, but most programs do not consider the nurses’ individual characteristics.

As mentioned above, none of the studies investigating the factors leading to resignation of newly graduated nurses consider the individual characteristics of nurses. As the individual characteristics of newly gradated nurses are important factors affecting their resignation, it is difficult to provide support without understanding those characteristics.

In this study, we focused on the characteristics that influence the early resignation of newly graduated nurses.

Understanding the characteristics of individuals prone to early resignation can help in planning appropriate interventions, follow-up, training, and labor management to reduce early resignation. Additionally, it can enable newly graduated nurses to become more self-aware and make changes to enhance their work experience.

Therefore, the purpose of this study was to compare the characteristics of newly graduated nurses upon employment with those who left and did not leave work within 1 year to identify strategies to prevent early resignation among newly graduated nurses based on their characteristics.

## Methods

### Sampling and sample size

This study conducted at a university hospital (acute care hospital) from 2018 to 2020 investigated the individual characteristics of newly graduated nurses and determined the characteristics that likely led to their early resignation.

Before starting the study, one of the authors explained the study design in person to a gathering of newly graduated nurses, requesting those who agreed to participate to submit consent forms. A total of 353 newly graduated nurses consented to participate.

### Data collection tool and method

Self-administered questionnaires were used to determine the characteristics of newly graduated nurses when they began working. The questionnaires used in this study were as follows.

**Fig. 1:**
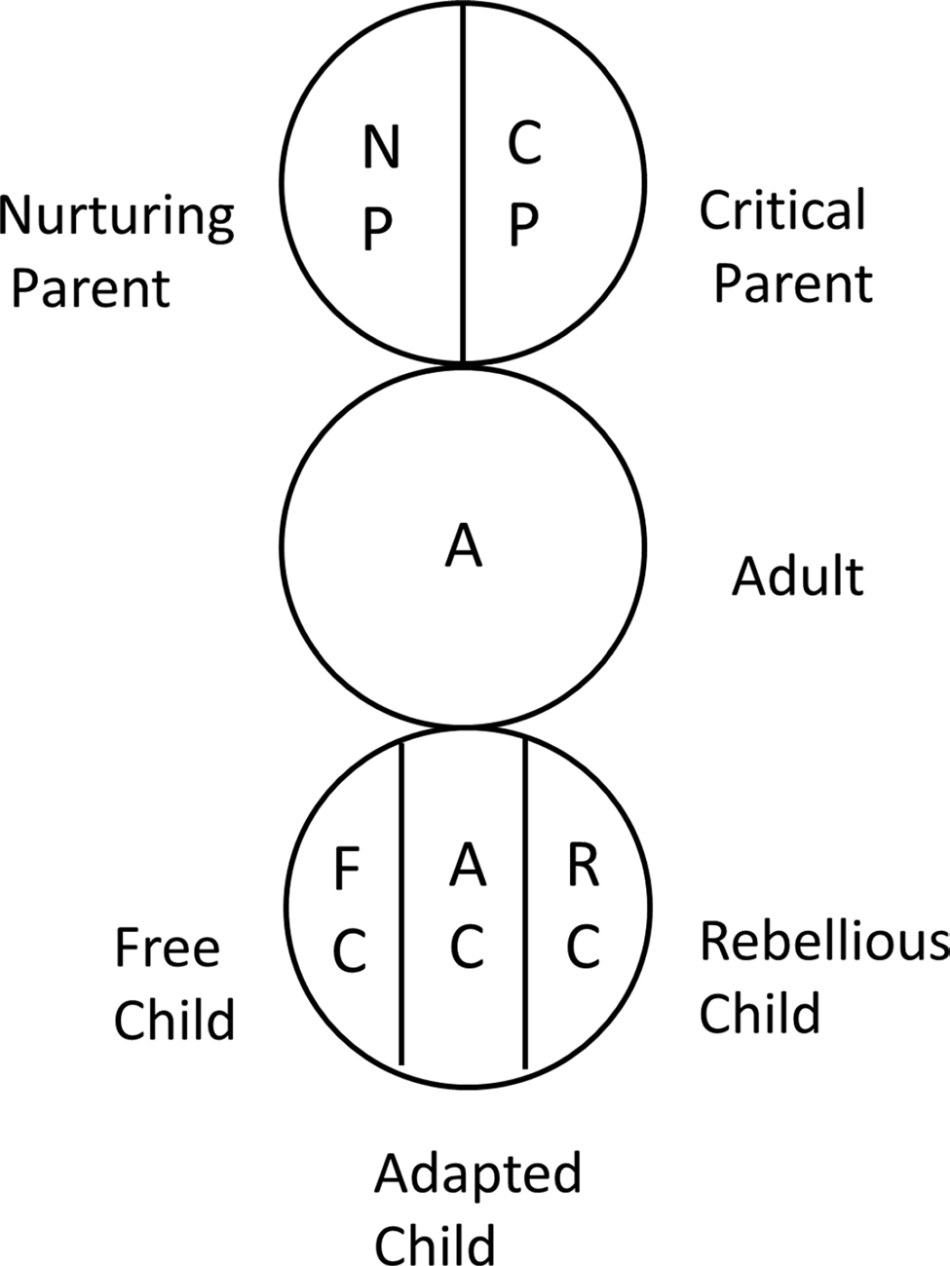
Fundamental concept of five egos

To assess the personality and interpersonal character of each participant, a Transactional analysis (TA) was conducted [16]. TA is widely used in education, business, medicine, nursing care, child-rearing, as and promoting mental health in the workplace and stimulating communication [17]. Therefore, in this study, we adopted the TA, a psychological theory proposed by Berne [18]. An egogram, used to evaluate personality traits, is a psychological scale theoretically based on the TA [19]. According to this theory, each person has three ego states: Parent, Adult, and Child. The parent ego consists nurturing and critical elements, and the child ego is divided into adapted and free elements, resulting in five functional ego states: Critical Parent (CP), Nurturing Parent (NP), Adult (A), Free Child (FC), and Adapted Child (AC) [20]. Typically, an egogram comprises these five factors [21]. However, in this study, we included the rebellious child (RC) factor in child ego [22]. Therefore, the child ego has three ingredients (Fig. 1). In terms of interpersonal relationships, we used Life Position, a central concept in TA, and a person’s convictions about the worth of the self and others [23]. The TA classifies possible life positions or psychological positions: “I am OK-You are OK (positive self and others),” “I am OK-You are not OK (positive self, negative others),” “I am not OK-You are OK (negative self, positive others),” and “I am not OK-You are not OK (negative self and others)” [23, 24]. The TA PACK SYSTEM^®^ developed by the Human skill Development Center Inc. (Tokyo, Japan) was used in this questionnaire. The scale has a high reliability, with an average Cronbach’s alpha of 0.82 and an average test-retest score of 0.91 [25].

The Japanese version of the State-Trait Anxiety Inventory (STAI-JYZ) [26, 27] was used to assess anxiety. STAI is recommended as an instrument to measure anxiety level [28]. It consists of 20 items for assessing trait anxiety and is rated on a 4-point scale [27, 29]. Trait anxiety levels were categorized into five grades (1–5) according to their scores [30]. Higher scores indicate elevated levels of anxiety. A score of 4 or 5 was defined as indicating high anxiety levels, as per the manual.

**Fig. 2:**
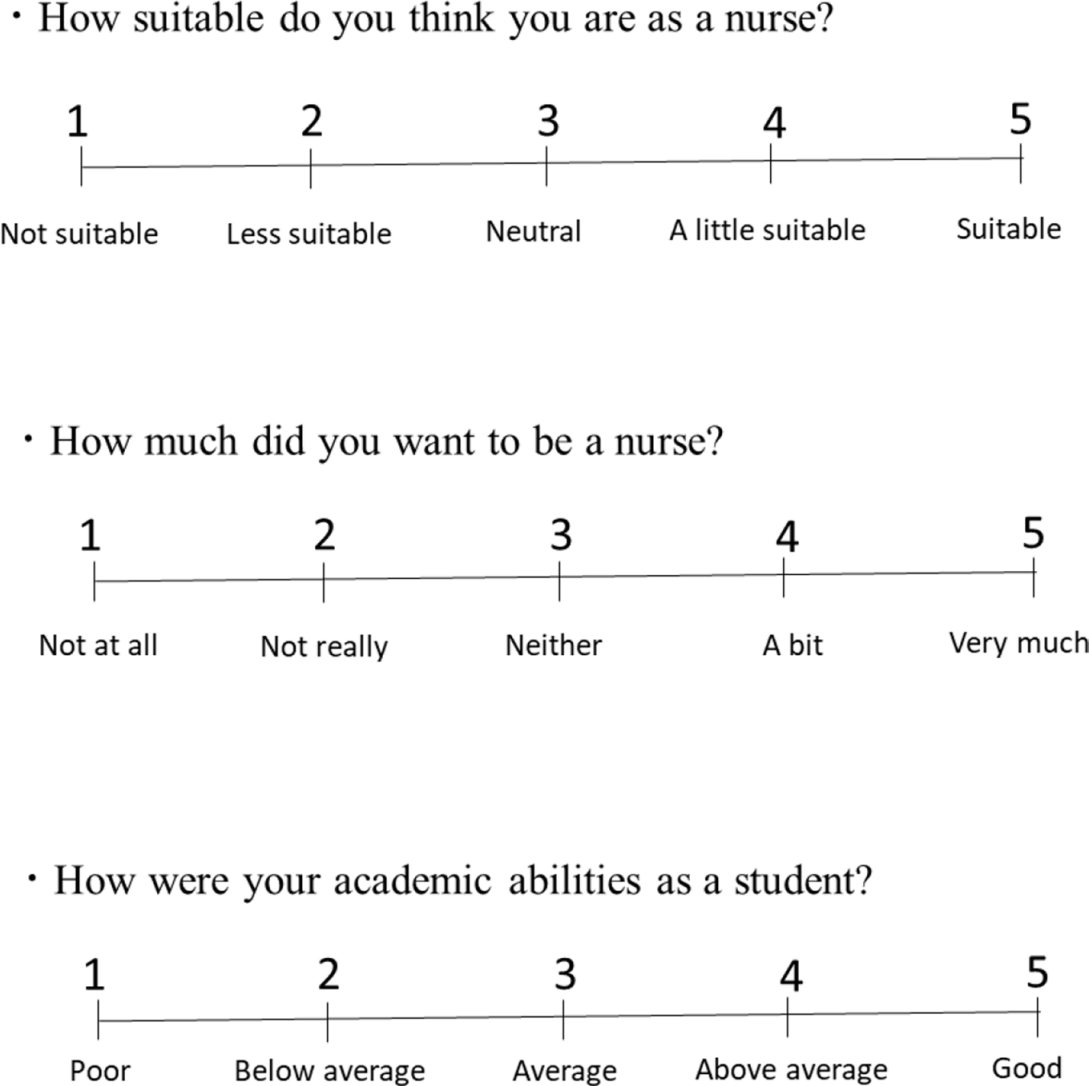
Questionnaire on nurse orientation, desire to be a nurse, and academic ability at school

Moreover, awareness of nurse orientation, interest in becoming a nurse, and self-perceived academic ability in school were investigated using a visual analog scale (VAS) (0–5) (Fig. 2). Higher scores corresponded to stronger orientation towards nursing (nurse orientation), desire to pursue nursing (desire), and academic proficiency (academic ability level). We considered 4 or 5 to reflect high levels, in line with the STAI.

### One-year observation

One-year observations were carried out on an annual basis. Those who decided to resign were interviewed by managers and/or the occupational health physician, and the factors leading to the nurses’ resignation were investigated. The semi-structured interviews helped in detecting the factors that led to resignation in detail. Cases in which retirement was unavoidable, such as due to relocation, marriage, or job change, were excluded.

At the end of the first year, the participants were categorized into those who resigned (case group) and did not resign (control group) in one year, and their questionnaires scores at the time of hiring were compared.

**Fig. 3:**
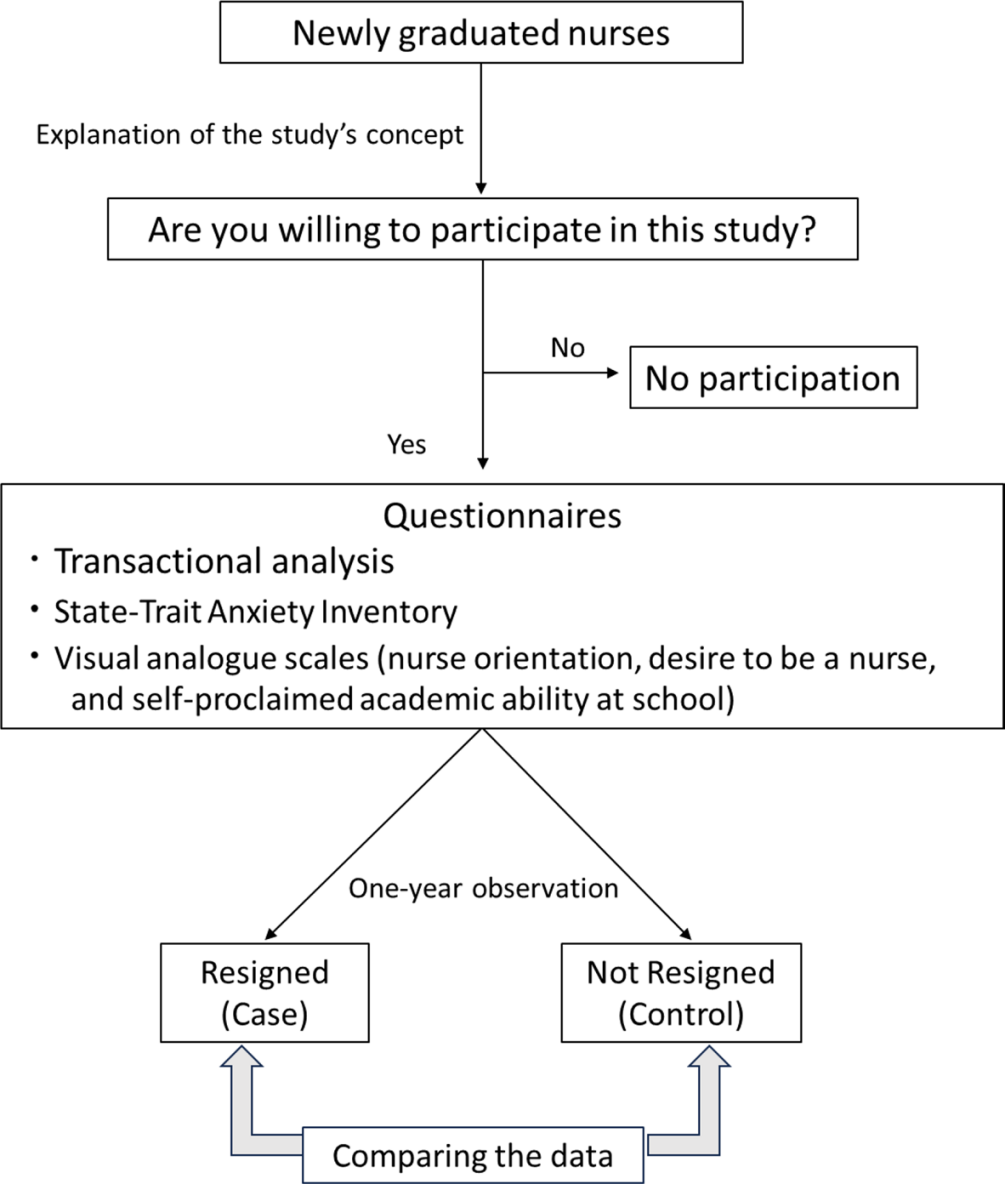
Study protocol of one year observation

The protocol of this study is summarized in Fig. 3.

### Statistical analyses

A chi-square test was used to compare the characteristics of nurses between the case and control groups. A logistic regression model was used to calculate the odds ratio (OR) with 95% confidence interval (CI) for resignation within one year associated with life position (I am OK; I am not OK; I am Neutral; You are OK; You are not OK; and You are Neutral), anxiety level, nurse orientation, desire to be a nurse, and academic ability level as independent variables.

Software program JMP Pro 17 (SAS Institute, Cary, NC, USA) was used for all analyses, setting the statistical significance at *p* < 0.05.

### Ethical considerations

The study was explained to all new employees in person, and only those who consented to participate answered the self-administered questionnaires and be followed for one year. This study received approval from the Ethics Committee of Jichi Medical University (approval no. 20–173).

### Confidentiality and anonymity

The survey was conducted through a questionnaire format, and to ensure confidentiality, all information was securely handled by an occupational health physician, with the data solely utilized for the purpose of this study.

## Results

Of the 353 participants, 32 nurses were from the case group and 304 were from the control group; 17 participants were eliminated owing to lack of data. The age distribution of the case and control groups was 22–23 and 21–29, respectively. All the case group participants were women, while the control group included 13 men.

As regards Egogram, the majority (21 (66%)) in the case group showed the following characteristics: (1) in the P factor, representing “parent ego,” CP < NP; (2) in the C factor, representing “child ego,” AC > FC and AC > RC. In contrast, 192 (63%) participants in the control group showed the same tendency.

Excluding Egogram scores, the other characteristics (life position, trait anxiety, nurse orientation, desire to be a nurse, and academic ability) of the participants in the case and control groups are summarized in Table 1.

**Table 1:** Life position, trait anxiety, nurse orientation, desire to be a nurse, and academic ability level in case and control groups

Life position							Case		Control		*p* value*				
	Case		Control			Trait anxiety level									
I am not OK-You are OK	17	(53%)	107	(35%)		High (STAI score ≧ 4)	22	(69%)	106	(35%)	< 0.05				
I am not OK-You are not OK	4	(13%)	20	(7%)											
I am not OK-You are Neutral	2	(6%)	12	(4%)		**Nurse orientation**									
I am Neutral-You are OK	2	(6%)	16	(5%)		Low (self score ≦ 2)	13	(41%)	56	(18%)	< 0.05				
I am Neutral-You are not OK	2	(6%)	3	(1%)											
I am OK-You are OK	4	(13%)	121	(40%)		**Desire to be a nurse**									
I am OK-You are not OK	1	(3%)	15	(5%)		Low (self score ≦ 2)	6	(19%)	20	(7%)	< 0.05				
I am OK-You are Neutral	0	(0%)	10	(3%)											
						**Academic ability level**									
I am OK	5	(14%)	146	(48%)		Not low (self score ≧ 3)	26	(81%)	255	(84%)	0.70				
I am not OK	23	(72%)	139	(46%)											
I am Neutral	4	(13%)	19	(6%)											
You are OK	23	(72%)	244	(81%)											
You are not OK	7	(22%)	38	(13%)											
You are Neutral	2	(6%)	10	(3%)											

*A chi-square test was performed for the comparison with case and control groups

The most common life position in the case group was “I am not OK-You are OK” (17 (53%)). While 23 (72%) participants held the position “I am not OK” for factor “I am,” 23 (72%) participants held the position “You are OK” for factor “You are.” STAI scores showed high trait anxiety, with 22 (69%) participants showing grade 4 or 5 trait anxiety levels. Furthermore, 13 (41%) participants showed low (1 or 2) nurse orientation, six (19%) showed a low desire to be a nurse, and 26 (81%) showed self-proclaimed academic ability levels of 3 or above.

Life positions varied more in the control than case group. Of the 304 participants, 121 (40%) held the “I am OK-You are OK” and 107 (35%) held the “I am not OK-You are OK” position. While 48% of participants held the “I am OK” and 46% of participants held the “I am not OK” position in the “I am” factor, 244 (80%) participants held the “You are OK” and only 38 (13%) held the “You are not OK” position in the “You are” factor. Both the “I am” and “You are” factors show significant differences between the case and control groups. STAI scores showed significantly lower trait anxiety in the control group, with only 106 (35%) participants having grade 4 or 5 anxiety levels. While 56 (18%) participants showed low (1 or 2) nurse orientation, 20 (7%) showed a low desire to be a nurse and 255 (84%) showed self-proclaimed academic ability levels of 3 or above. Significant differences were found in nurses’ orientation and desire to be a nurse between the case and control groups.

Egogram scores of most participants showed CP < NP, AC > FC, and AC > RC. Therefore, we combined the other factors with the Egogram scores. Of the case group participants who showed CP < NP, AC > FC, and AC > RC, 15 (71%) held the “I am not OK” (negative self) position, 15 (71%) showed strong trait anxiety, and 12 (57%) showed strong negative self and trait anxiety. Furthermore, while 11 (52%) participants showed low nurse orientation, one (5%) showed a low desire to be a nurse. In contrast, of the control group participants who showed CP < NP, AC > FC, and AC > RC, 107 (56%) held the “I am not OK” (negative self) position, 81 (42%) showed strong trait anxiety, and 57 (30%) showed negative self and strong trait anxiety. Furthermore, 32 (17%) participants showed low nurse orientation and seven (4%) participants showed a low desire to be a nurse. Participants with CP < NP, AC > FC, and AC > RC showed significant differences between the case and control groups in the following factors: (1) negative self, (2) strong trait anxiety, (3) strong trait anxiety and negative self, and (4) low nurse orientation. The control group showed a significantly lower percentage of participants with these characters than the case group (Table 2).

**Table 2:** Comparison between case and control groups, in combination of three factors with other factor(s)

Factors		Case			Control		*p* value*
3 factors + Negative self		15	(71%)		107	(56%)	< 0.05
3 factors + Strong trait anxiety		15	(71%)		81	(42%)	< 0.05
3 factors + Negative self +Strong trait anxiety		12	(57%)		57	(30%)	< 0.05
3 factors + Low nurse orientation		11	(52%)		32	(17%)	< 0.05
3 factors + Low desire to be a nurse		1	(5%)		7	(4%)	0.80

3 factors: CP < NP, AC > FC and AC > RC

*A chi-square test was performed for the comparison with case and control groups

Table 3 presents the logistic regression analysis results of those with CP < NP, AC > FC, and AC > RC. It presents the crude and adjusted OR of nurses’ resignation within one year of gaining employment for each value listed in Table 1.

**Table 3:** Association between resignation within one year with life position, anxiety level, nurse orientation, desire to be a nurse, and academic ability level

Factor	Crude OR (95% CI)	*p* value*		Adjusted OR (95% CI)	*p* value*
I am OK	0.26 (0.10–0.66)	< 0.05		0.40 (0.13–1.16)	0.09
I am Neutral	1.33 (0.41–4.28)	0.63		1.57 (0.41–6.01)	0.51
I am not OK	1 (Reference)			1 (Reference)	
You are OK	0.51 (0.21–1.27)	0.15		0.44 (0.16–1.22)	0.12
You are Neutral	0.49 (0.09–2.59)	0.40		0.28 (0.04–1.91)	0.20
You are not OK	1 (Reference)			1 (Reference)	
Strong trait anxiety	4.11 (1.88–9.00)	< 0.05		2.55 (1.00–6.48)	< 0.05
Low nurse orientation	12.32 (5.18–29.30)	< 0.05		24.12 (6.57–88.48)	< 0.05
Low desire to be a nurse	1.33 (0.52–3.41)	0.56		0.69 (0.12–4.06)	0.69
Low academic ability	1.20 (0.47–3.07)	0.70		0.21 (0.04–1.28)	0.09

*The result of Logistic regression analysis

OR = odd ratio, CI = confidence interval

Our crude analysis shows a significant relationship between the newly graduated nurses’ characteristics of “I am OK” (positive self), strong trait anxiety, and low nurse orientation and their resignation within one year of gaining employment. Our adjusted analysis also shows a significant relationship between the nurses’ strong trait anxiety and low nurse orientation and their resignation within one year.

## Discussion

As human resources are an important factor in all occupational fields [31], it is important to prevent the early resignation of new employees. However, determining in advance the employees likely to resign is not an easy task. While NIOSH suggests that job stress can lead to harmful physical and emotional responses, individual (age, sex, marital status, character, self-estimation, etc.) and non-work-related (family demands) and buffering (social support from supervisors, coworkers, and family) factors can also cause stress reactions [14, 15]. Tei-Tominaga reported psychological response as one of the risk factors affecting the resignation of newly graduated nurses [32]. Thus, employees’ characteristics and self-estimation are factors related to their early resignation.

This study investigated the characteristics of newly graduated nurses to determine those that tended to lead to early resignation.

We found no difference in characteristics between the case and control groups based on Egogram scores. However, there were significant differences between the case and control groups in other characteristics. Specifically, newly graduated nurses having the following characteristics tended to resign early: (1) negative self, (2) strong trait anxiety, (3) strong trait anxiety and negative self, and (4) low nurse orientation.

Every individual has one of the following basic life positions: “I am OK, you are OK” (positive self and others), “I am OK, you are not OK” (positive self, negative others), “I am not OK, you are OK” (negative self, positive others), and “I am not OK, you are not OK” (negative self and others) [33]. The “I am not OK-You are OK” position is common for individuals who feel powerless. Such individuals tend to withdraw from studies and experience depression. Moreover, people with the “I am not OK-You are not OK” position tend to feel bad about themselves, have no confidence, and do not trust others [34]. Therefore, those with the “I am not OK” position have a self-deprecating personality and do not accept themselves, increasing their stress [35]. Thus, an individual with “I am not OK” position may also experience mental health problems leading to resignation.

A relationship exists between trait anxiety and depression [36, 37]. Individuals with anxiety or depressive disorder display significantly elevated trait anxiety. Moreover, anxiety and depressive symptom severities are strongly correlated with trait anxiety. Trait anxiety is a non-specific measure of negative affectivity [37]. A study on nursing students found that severe anxiety affected physical and mental health [38]. These results support our findings that strong trait anxiety can affect the mental health of newly graduated nurses and lead to their early resignation.

Although the case and control groups showed no significant difference in the nurses’ desire to be a nurse and self-proclaimed academic ability at school, a significant difference existed between them in work orientation. Positive work orientation is related to coping with burnout and depression [39, 40]. Therefore, those with low nurse orientation may choose early resignation because they find no motivation.

Our logistic regression analysis shows that strong trait anxiety and low nurse orientation are significantly related to the early resignation of newly graduated nurses. Moreover, “negative self” has no influence on early resignation, although its crude OR was 0.26 (0.10–0.66). Therefore, self-deprecating personality can have some effect on early resignation. To prevent the resignation of newly graduated nurses, their education must focus on reducing anxiety and helping them find meaning in work.

This study had some limitations. First, we conducted the study at a university hospital. Although we planned to involve several hospitals, we could not do so owing to COVID-19-related restrictions. Therefore, the case group in our study was small and the participants could have been biased. Second, we used visual analogue scales to investigate the nurses’ awareness of nurse orientation, desire to be a nurse, and self-proclaimed academic ability at school. We could find no objective measurement methods for these factors. VAS is a simple but valuable instrument to measure a characteristic or attitude that cannot be easily measured directly [41]. Therefore, we adopted VAS, although there were some concerns about its validity. Third, although VAS is valuable in estimating the changes within individuals, it cannot compare individuals at a single time point [42]. Therefore, the VAS values may differ between individuals.

Future studies need to find better ways to prevent the early resignation of newly graduated nurses.

## Conclusion

Most newly graduated nurses tended to have CP < NP, AC > FC, and AC > RC characteristics. Of these, strong trait anxiety and low nurse orientation significantly affected the early resignation of nurses. The nurses’ self-deprecating personality also showed some effect on their early resignation.

From the results of this study, the early resignation of nurses can be prevented by understanding their characteristics at the time of employment and implementing early intervention, such as education programs to reduce anxiety, and helping those with strong trait anxiety and low nurse orientation to find meaning in their work.

## Data Availability

The datasets used and/or analyzed in the current study are available from the corresponding author upon reasonable request.

## Abbreviations

NIOSH: The National Institute for Occupational Safety and Health
TA: Transactional analysis
CP: Critical parent
NP: Nursing parent
FC: Free child
AC: Adapted child
RC: Rebellious child
STAI: State-Trait Anxiety Inventory
